# Lessons From COVID-19: Physical Exercise Can Improve and Optimize Health Status

**DOI:** 10.3389/fmed.2022.834844

**Published:** 2022-05-13

**Authors:** Dario Cerasola, Christiano Argano, Salvatore Corrao

**Affiliations:** ^1^Department of Psychology, Educational Science and Human Movement, University of Palermo, Palermo, Italy; ^2^Department of Internal Medicine, National Relevance and High Specialization Hospital Trust ARNAS Civico, Di Cristina, Benfratelli, Palermo, Italy; ^3^Dipartimento di Promozione della Salute, Materno Infantile, Medicina Interna e Specialistica di Eccellenza “G.D'Alessandro”, PROMISE, University of Palermo, Palermo, Italy; ^4^Internal Medicine COVID-19 Unit, National Relevance and High Specialization Hospital Trust ARNAS Civico, Di Cristina, Benfratelli, Palermo, Italy

**Keywords:** physical activity, post COVID-19, health status, mental status, life style

## Abstract

The outbreak of Coronavirus Disease 2019 (COVID-19) has caused increasing challenges for healthcare systems globally. The disease spread rapidly from Wuhan to the rest of the world, involving more than 400 million individuals and including more than 5 million deaths. In dealing with the pandemic, China and other countries took protective measures such as promoting social distancing, canceling public gatherings, closing schools, quarantining, and imposing lockdowns. All these measures lead to physical inactivity. Being physically inactive has significant repercussions on the status of physical and mental wellbeing, and it is associated with anxiety, stress, increased chronic disease risk, and worsening of chronic conditions. In this sense, the relevance of maintaining a healthy lifestyle through physical exercise has been outlined by the World Health Organization (WHO). The aim of this mini review is to discuss the importance of physical activity in the context of the COVID-19 pandemic, highlighting the benefits of physical activity and exercise that could be potentially effective treatment strategies for comorbid chronic conditions, long covid syndrome (LCS), and symptoms such as depression and anxiety.

## Introduction

In December 2019, a novel coronavirus named severe acute respiratory syndrome coronavirus 2 (SARS-CoV-2) caused an infectious disease—“coronavirus disease 2019” (COVID-19)—that spread aggressively across the globe. This infection can be asymptomatic or be associated with mild to moderate different symptoms and clinical manifestations ranging from fever, dry cough, and shortness of breath to interstitial pneumonia and acute respiratory distress syndrome (ARDS), requiring hospitalization in more severe cases (1). Different countries took protective measures such as promoting social distancing and traveling restrictions, canceling public gatherings, closing schools, quarantining, and imposing lockdowns to contain the outspread of the virus (2). These restrictions had a negative effect on people's lifestyles. COVID-19 has influenced personal relationships, the educational process, eating habits, and the way of practicing exercise, favoring a sedentary lifestyle as well as the consumption of qualitatively unhealthy diets, thus exposing people to an obesogenic environment (3). Firstly, prolonged self-isolation can adversely affect the psychological response, facilitating post-traumatic stress symptoms, and anxiety (4). Secondly, quarantine and lockdowns lead to physical inactivity, which contributes to negative health consequences such as obesity, premature aging, cardiovascular vulnerability, bone loss, decreased aerobic capacity, and musculoskeletal atrophy (5). Moreover, decreased physical activity has a negative impact on the management of chronic diseases such as cardiovascular diseases, type 2 diabetes, obesity, and malignancies (6). Different studies showed the positive effect of physical activity on health status. In particular, exercise is able to prevent metabolic disorders, cardiovascular and pulmonary diseases, and muscle, bone, and joint diseases (7–10), and the latest recommendations emphasize the need for symptom-titrated physical activity and tailored exercise in rehabilitation for mitigating the post-covid-19 syndrome. In light of the above reasons, the aim of this review is to explore the role of physical activity during the COVID-19 pandemic, outlining its benefits as a potential treatment for health status, chronic conditions, and Long Covid Syndrome and providing practical recommendations.

## Changes In Lifestyle: Health Status

Imposed radical changes in lifestyle induce a radical transformation of habits, including regular physical activity and routine daily activities, with an inevitable social detachment that implies negative effects on mood and mental health (11).

Anxiety, stress, and psychological fear related to covid-19 and its associated restrictions make it difficult to spend extended periods of time confined to the same four walls. People might experience sadness, loneliness, worries about family, changes in sleep or eating patterns, difficulty sleeping or concentrating, worsening of chronic health diseases, and increased use of alcohol, tobacco, drugs, and food (12), and among these there is also the possibility of obsessive-compulsive actions, such as repeated temperature measurement (13).

Moreover, there is less time spent performing normal daily activities, and the perception of time changes so it seems to go slower; this is caused by prolonged homestay and may lead to an increased sedentary lifestyle, such as spending excessive time sitting and reclining, increased screen activities (using a mobile device, playing games and chatting, and watching television), a reduction in regular physical activity (hence lower energy expenditure), or increasing eating. These behaviors can determine negative consequences, including increased risk and/or potential for worsening health conditions (13), including the reduction of muscle mass and strength, which can be further exacerbated in those people with obesity (14, 15). To help to manage and reduce this strong psychological stress, the WHO has issued international guidelines (16, 17).

## Physical Exercise Benefits

In this regard, physical activity promotes good health status and better quality of life. Regular moderate and vigorous physical activity reduces the risk of many adverse health outcomes in all age groups and in persons with chronic conditions or disabilities (Table 1). Physical inactivity affects the cardiovascular, metabolic, respiratory, and neuromuscular systems (18) and is associated with fat deposition and low-grade systemic inflammation. Subjects with comorbidities such as hypertension (19), diabetes (20), obesity (21), and heart and respiratory diseases (22) are characterized by low-grade chronic inflammation, which may account for, in a proportion of subjects who are persistently inflamed, significantly higher rates of hospitalization and mortality and poorer prognosis with coronavirus infection (23–25). In addition, these conditions are often related to poor eating habits, sedentary patterns, and physical inactivity (26). Indeed, a baseline sedentary lifestyle is an independent risk factor for mortality in hospitalized patients with COVID-19 (27). Regarding hypertension, physical exercise has been proposed as one of the main strategies to reduce blood pressure as an alternative to pharmacologic therapies (28). In this sense, training programs performed at moderate intensity three times per week seem to be optimal to reduce blood pressure (29). Concerning metabolic disease diabetes significantly increases the risk of hospitalization and death in COVID-19 patients (30). In this sense, exercise is an important treatment strategy to improve long-term glycemic control in people with type 2 diabetes (31). The benefits of exercise for glycemic control are largely explained by an increase in whole-body insulin sensitivity. Regarding the type of exercise, resistance and aerobic exercises are both recommended as effective treatments for people with diabetes (32). In addition, visceral obesity represents one of the strongest predictors of hospitalization in COVID-19 patients and intensive care units (33, 34). Visceral adipose tissue increased systemic and local inflammation (35) due to the enhancement of expression of pro-inflammatory cytokines (36), determining health status changes (37–39). Aerobic training combined with resistance exercise training is indicated as a program to improve systemic inflammation with weight loss (40). This also applies to childhood obesity. In fact, physical inactivity has been identified as a risk factor along with biological, psychosocial, and behavioral aspects (41). Adolescents who did less physical activity were more likely to be overweight or obese and less likely to have strong prior physical activity habits (42).

**Table 1:** Principal physical activity benefits related to different age groups.

**Children and youth**	**Adults and older adults**
Improved bone and muscular health Improved weight status	Lower risk of cardiovascular disease
	Lower risk metabolic diseases Improve blood lipid profile
Improved cardiorespiratory and muscular fitness	Lower risk of cancers
Improved cognition and neurological functions Improved socialization	Reduced risk of dementia and neurological diseases Improved quality of life
	Reduced anxiety
	Reduced risk of depression and anxiety
	Improved sleep
	Slowed or reduced weight gain Weight loss
	Improved bone health lmproved muscular function Lower risk of falls
	Lower risk of fall-related injuries

Physical activity can improve cognition and cardiorespiratory and muscular fitness and can also reduce the risk of depression in young men. Moreover, physical exercise is included in some preventive programs (43–45). Aerobic, resistance physical activity, balance, and stretching exercises are beneficial. For most people, health benefits are obtained when individuals performed at least 150 min of moderate-intensity physical activity, but additional benefits occur as the amount of physical activity increases through higher intensity, greater frequency, and/or longer duration.

Exercise is not only good for physical health; it also supports emotional and mental health. Certainly, exercise improves physical health and physique, but that is not what motivates most people to stay active. People who perform regular physical activity are inclined to do so because it gives them an improved sense of wellbeing (46). Different studies showed the psychological benefits of regular physical activity on principal disorders such as anxiety, depression, and stress, with a similar effect to pharmacological treatment (47–49) In particular, physical activity can help reduce anxiety and improve mild to moderate symptoms of depression. For example, regular running or walking for 15–20 min or more reduces the risk of major depression by 26% and relapse prevention (50). Physical exercise, with its natural effect, represents an effective anti-anxiety treatment, relieves nervous and muscular tension, improves mental energy, and enhances wellbeing through the release of endorphins (50, 51). People under stress commonly complain of pain in the neck muscles, shoulder and back in tension, irregular heart rates, chest tightness, and headaches. The discomfort of all these physical symptoms can in turn lead to even more stress, creating a vicious cycle between mind and body (50, 52). The bad mind–body connection might be discontinued by physical exercise, which relaxes the muscles and relieves tension, providing hormones to help the brain (52). In addition, people who are physically active sleep better. In fact, moderate or vigorous physical activity is associated with less time to fall asleep, improved sleep quality, and a significant reduction in daytime sleepiness. The psychological benefits of physical activity are due to the interaction of four elements that must be mentioned: distraction, self-efficacy, rejuvenation, and physiological change (50). Distraction is considered as the elimination of negative thoughts; self-efficacy is how feelings play a protective role against stress and anxiety; rejuvenation refers to an increase in energy level and a reduction of nervous tension; physiological change refers to modifications of the neurological, cardiovascular, mental, respiratory, muscle, skeletal, and digestive systems during and after exercise (52, 53).

Exercise is a powerful factor to counteract mental disorders because it affects the brain by the release of neurotrophic growth factors and functional and structural changes involving the prefrontal cortex and hippocampus. It reduces inflammation and promotes feelings of calm and wellbeing.

Moreover, exercise stimulates the production of powerful chemicals like endorphins in the brain, which makes us feel good. Finally, exercise can also serve as a distraction and can interrupt the cycle of negative thoughts and worries that feed mental problems. For adults, the American College of Sport Medicine (ACSM) (7) recommends aerobic activity at moderate intensity for ≥30 min·d^−1^ on ≥5 d·wk^−1^ for a total of ≥150 min·wk^−1^ and resistance exercises for each of the major muscle groups, and balance, agility, and coordination exercises and flexibility training for each of the major muscle-tendon groups (total of 60 s per exercise) on ≥2 d·wk^−1^ (recommended 2–3 d·wk^−1^). In addition, the WHO (8) recommends that children and youths should perform an aerobic activity at moderate/vigorous intensity ≥60 min·d^−1^ every day with exercise to strengthen muscle ≥3 d·wk^−1^. Adults aged 18–64 years old should do moderate-intensity aerobic physical activity for a total of ≥150 min·wk^−1^ or aerobic physical activity for at least 75 min·wk^−1^ while people aged 64 years or older should perform aerobic activity at moderate intensity for a total of ≥150 min·wk^−1^ or aerobic physical activity at least 75 min·wk^−1^ of vigorous intensity.

Resistance training should be done to improve balance and fall prevention involving major muscle groups at least ≥2 d·wk^−1^.

Exercise and physical activity at home are always possible and easily implementable; this includes cardiovascular activity, strength exercises, balance exercises, and stretching. Nowadays, technologies help us through many e-systems. The use of information and communication technology, such as exercise videos, mobile apps, and social media, to support health and healthcare (called “e-Health”) are new strategies for maintaining physical function and mental health during this period (54). Otherwise, examples of home training exercises include stair climbing, skipping, sitting up and down on a chair, squats, push-ups, core stability exercises, isometric exercises, home walking, and strength exercises with household items or, alternatively, Yoga (55) or Tai Chi (56) activities.

The main goal should be to perform a combination of moderate and vigorous-intensity activity or at least 20 min^.^d^−1^ of vigorous exercise or at least 30 min^.^d^−1^ of moderate physical exercise. In addition, strength exercise with a combination of aerobic activity is the optimum strategy. Physical activity must include balance, control, and stretching exercises.

## Sport and Covid-19 Transmission

Even if the coronavirus emergency has not ended, the resumption of physical activity is important. However, the circulation of the virus in the population is still present, and outdoor exercise thus offers a series of preventive methods.

The airborne transmission of SARS-CoV-2 has been demonstrated to be higher than SARS-CoV-1, considering that the virus remains suspended and infectious for hours (57). Different studies showed that SARS-CoV-2 infection may occur not only over close distances (58); within indoor environments, small aerosol particles containing the virus may cover distances up to 10 meters from the emission sources (59). Moreover, many countries have required people to maintain a critical interpersonal distance above 1.5 m to limit the spread of SARS-CoV-2 infection. This “interpersonal distance” is considered important and effective because it is assumed that most of the droplets indeed fall down and reach the floor and/or evaporate before having traveled a distance of 1.5 m. However, micro-droplets have very little inertia, and when two people are walking or running in each other's vicinity, even at a 1.5 m distance, due to the airflow patterns and people's movements, these micro-droplets could be transferred from person to person.

Blocken et al. (60) showed how exposure to droplets occurs when a trailing runner is positioned in the slipstream of the leading runner, though the authors did not consider the effect of the other's wind direction. In particular, substantial droplet exposure occurs when the trailing runner is positioned in the slipstream of the leading runner, up to a distance between them that depends on the traveling speed. Walking at a speed of 4 km/h for a distance of about 5 m leads to no droplets reaching the upper torso of the trailing runner. Running at a speed of 14.4 km/h, this distance is about 10 m.

Finally, taking into account outdoor sports, such as cycling, the distance should be increased as the speed increases (for cycling, consider the greater speed compared to the runners). Further studies should analyze the dynamics of diffusion in other outdoor sports for two reasons: to take into account the dynamics of the air flows, and to consider the better range of droplets in relation to the greater strength and frequency of exhalation in subjects who play sports. In conclusion, the daily target indoor exercise should consist of a balance between cardiovascular and strength exercises at moderate and vigorous intensities and should include stretching and balance exercises. Moreover, considering that micro-droplets have very little inertia, during outdoor sports, such as running and cycling, athletes with little interpersonal distance can be exposed to a flow of droplets. Therefore, it is necessary to increase the interpersonal distance relative to the type of sports. It is worth noting that physicians must be able to prescribe physical exercise providing detailed indications, particularly to subjects with chronic conditions. In addition, clinicians must be able to provide information about the importance of exercise as pharmacological treatment.

## Post Covid-19: Long Covid Syndrome

COVID-19 presents with different clinical manifestations, from fever, dry cough, and dyspnea to myocarditis, kidney disease, coagulation abnormalities, interstitial pneumonia, and acute respiratory distress syndrome (ARDS), which require hospitalization and intensive care (61–64). While the majority of infected subjects recover within 2–3 weeks, some people present persistent symptoms irrespective of age and underlying health condition, and this is referred to as “Long Covid Syndrome” (LCS). LCS is a term to describe the effects of Covid-19 that continue for weeks or months beyond the initial SARS-CoV-2 infection. LCS is characterized by the presence of signs and symptoms that continue for more than 12 weeks and are not explained by an alternative diagnosis. In particular, people with LCS showed impairment of the function and structure of multiple organs. The most important symptoms experienced by people affected by LCS are premature fatigue, dyspnea and shortness of breath, chest pain or tightness, difficulty sleeping (insomnia), heart palpitations, muscle and joint pain, depression and anxiety, diarrhea, stomach aches, and loss of appetite (65–68). A very recent study highlighted the importance of physical activity. Physical exercise is an effective therapeutic strategy to combat COVID-19 infection, mitigate the consequences of infection, and improve immunosurveillance. Nieman points out the presence of three prevention levels (69). The primary prevention level regards the role of physical activity as an immune system adjuvant in contrasting infectious diseases (70–73).

The secondary prevention level refers to the potentially positive role of physical activity in increasing COVID-19 vaccine efficacy, and the tertiary prevention level relates to the crucial role of physical training and rehabilitation, which can be directed to improve quality of life, health, and physical fitness. Indeed, prolonged immobilization is very common in people with COVID-19, causing a reduction of muscle functions, atrophy, and sarcopenia. Sarcopenia is associated with an increased risk of malnutrition, frailty, disability, falls, and loss of independence (74).

In this sense, recent studies showed the importance of nutritional strategies for the rehabilitation of COVID-19 patients. In particular, a three-step nutritional protocol was developed for patients with SARS-CoV-2 infection. The first step consists of nutritional assessment and malnutrition screening (multidisciplinary staff performed an anthropometric parameter, impedance and vectorial analysis, weight loss, hematochemical parameters, shallowing, and intake evaluation). The second step includes nutritional treatment (an adequate diet was recommended by a previous analysis, determined by total energy, protein, carbohydrate, lipid, and water requirements) while the third step involves continuous monitoring (the personal patient protocol was changed in relation to physiological parameters like body weight, total energy intake, blood tests, and clinical condition) (75). Otherwise, another approach involves a screening assessment (Malnutrition Universal Screening Tool, Reduced muscle mass, Eating Assessment Tool, Kennedy classification for masticatory function), nutritional interventions at admission and during hospital stay (dietary counseling and/or food modification, Essential Amino Acids supplementation and/or oral dysphagia product, and enteral or parenteral nutrition), and rehabilitation post Covid-19 (nutritional care plan, dysphagia rehabilitation, and rehabilitation of masticatory function) (76).

In addition, Udina et al. (77). suggested a 30-min daily multicomponent therapeutic exercise intervention, including 30-min multi-components exercises 7 days/week (i.e., resistance training with intensity between 30–80% of the repetition maximum and endurance aerobic training up to 15-min) and balance training for post-COVID-19 rehabilitation. This physical training represents a fundamental tool to ameliorate the functional status of patients with COVID-19, including subjects who required intensive care unit stay.

**Figure 1:**
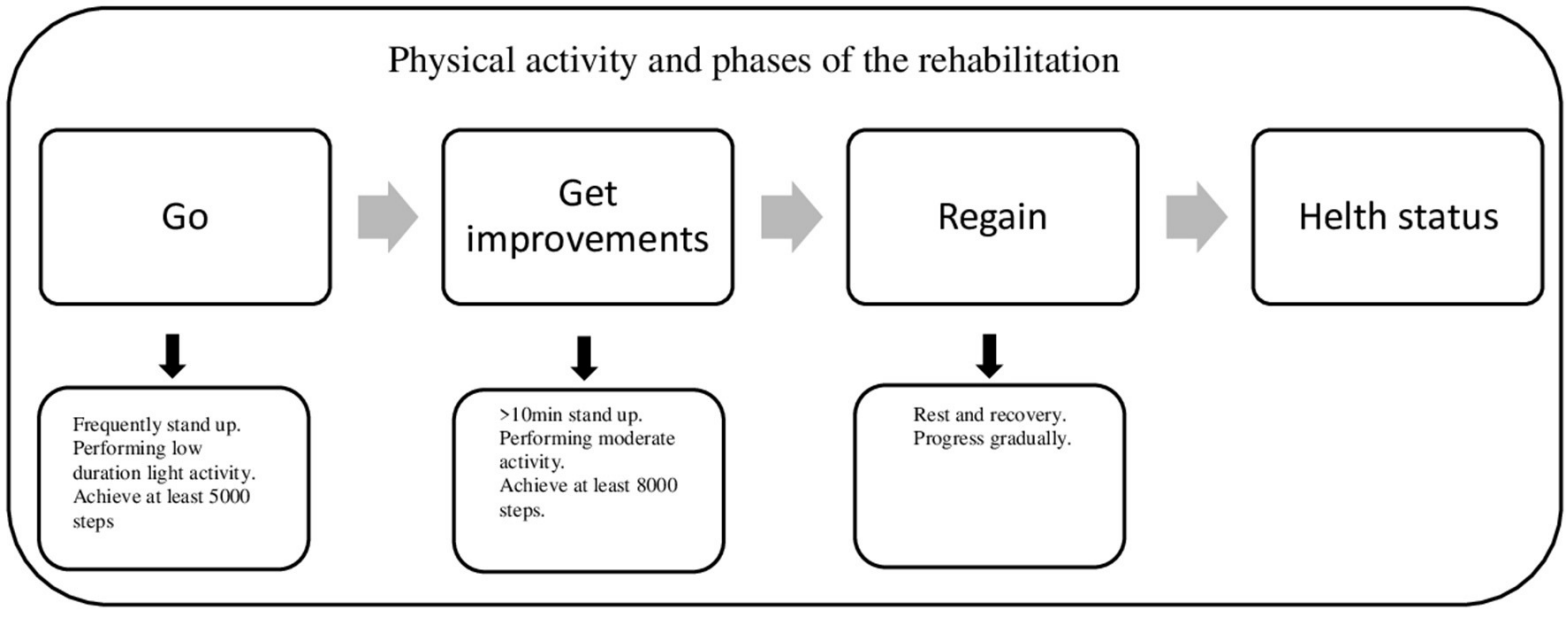
Steps of rehabilitation during long COVID syndrome.

Although symptoms of LCS are characterized by improvement and relapse phases, physical activity interventions normally improve the quality of life reducing the negative effect of LCS. Particularly, rehabilitation includes three steps that concern the return to health: go, get improvements, and regain (Figure 1). Go: In this step, people who suffer from LCS can initially find it difficult to re-engage with physical activity, and all movements are therefore important. This also includes the ability to make physical activity by developing programs tailored to the individual. Get improvements: in this phase, to achieve recommended physical activity levels, it is mandatory to start with lower intensity activity. This kind of exercise promotes health status by improving the baseline functional capacity. Regain: in the third step, the precarious state of health can result in a seesaw of physical conditions that can relapse unexpectedly, interfering with an individual's progress, increasing the risk of injury, and reducing the immune response.

## Conclusion

Physical activity must be a strategy of therapeutic action and not only an unstructured suggestion. Because the benefits of exercise and nutrition help physically and mentally, it is important to maintain a regularly active lifestyle for a healthy life during the pandemic and post COVID-19 crisis to prevent chronic diseases and LCS syndrome.

## Author Contributions

DC and SC designed the study. DC and CA performed the research and wrote the manuscript. SC and CA reviewed and edited the manuscript. All authors contributed to the article and approved the submitted version.

## Conflict of Interest

The authors declare that the research was conducted in the absence of any commercial or financial relationships that could be construed as a potential conflict of interest.

## Publisher's Note

All claims expressed in this article are solely those of the authors and do not necessarily represent those of their affiliated organizations, or those of the publisher, the editors and the reviewers. Any product that may be evaluated in this article, or claim that may be made by its manufacturer, is not guaranteed or endorsed by the publisher.
